# Preterm Birth, Small for Gestational Age, and Large for Gestational Age and the Risk of Atrial Fibrillation Up to Middle Age

**DOI:** 10.1001/jamapediatrics.2023.0083

**Published:** 2023-04-24

**Authors:** Fen Yang, Imre Janszky, Mika Gissler, Sven Cnattingius, Nathalie Roos, Maohua Miao, Wei Yuan, Jiong Li, Krisztina D. László

**Affiliations:** 1 Department of Global Public Health, Karolinska Institutet, Stockholm, Sweden; 2 Department of Public Health and Nursing, Norwegian University of Science and Technology, Trondheim, Norway; 3 Department of Knowledge Brokers, Finnish Institute for Health and Welfare, Helsinki, Finland; 4 Academic Primary Health Care Centre, Region Stockholm, Stockholm, Sweden; 5 Department of Molecular Medicine and Surgery, Karolinska Institutet, Stockholm, Sweden; 6 Division of Clinical Epidemiology, Department of Medicine Solna, Karolinska University Hospital, Karolinska Institutet, Stockholm, Sweden; 7 NHC Key Laboratory of Reproduction Regulation, Shanghai Institute for Biomedical and Pharmaceutical Technologies, Fudan University, Shanghai, China; 8 Department of Clinical Medicine-Department of Clinical Epidemiology, Aarhus University Hospital, Aarhus, Denmark; 9 Department of Public Health and Caring Sciences, Uppsala University, Uppsala, Sweden

## Abstract

**Importance:**

Adverse birth outcomes, including preterm birth, small for gestational age (SGA), and large for gestational age (LGA) are associated with increased risks of hypertension, ischemic heart disease, stroke, and heart failure, but knowledge regarding their associations with atrial fibrillation (AF) is limited and inconsistent.

**Objective:**

To investigate whether preterm birth, SGA, or LGA are associated with increased risks of AF later in life.

**Design, Setting, and Participants:**

This multinational cohort study included Danish, Swedish, and Finnish national health registries. Live singleton births in Denmark from 1978 through 2016, in Sweden from 1973 through 2014, and in Finland from 1987 through 2014, who were followed up until December 31, 2016, in Denmark, December 31, 2021, in Sweden, and December 31, 2014, in Finland were included. Data analyses were performed between January 2021 and August 2022.

**Exposures:**

Preterm birth (less than 37 gestational weeks), SGA (less than 10th percentile birth weight for gestational age), and LGA (more than 90th percentile birth weight for gestational age) identified from medical birth registers.

**Main Outcomes and Measures:**

Diagnosis of AF obtained from nationwide inpatient and outpatient registers. The study team ran multivariable Cox proportional hazard models and flexible parametric survival models to estimate hazard ratios (HRs) and 95% CIs for AF according to preterm birth, SGA, and LGA. Sibling analyses were conducted to control for unmeasured familial factors.

**Results:**

The cohort included 8 012 433 study participants (maximum age, 49 years; median age, 21 years; male, 51.3%). In 174.4 million person-years of follow-up, 11 464 participants had a diagnosis of AF (0.14%; median age, 29.3 years). Preterm birth and LGA were associated with increased AF risk in both the full population cohort and in the sibling analyses; the multivariate HRs from the cohort analyses were 1.30 (95% CI, 1.18-1.42) and 1.55 (95% CI, 1.46-1.63), respectively. Preterm birth was more strongly associated with AF in childhood than in adulthood. Children born SGA had an increased risk of AF in the first 18 years of life but not afterwards.

**Conclusions and Relevance:**

Preterm births and LGA births were associated with increased risks of AF up to middle age independently of familial confounding factors. Individuals born SGA had an increased AF risk only during childhood.

## Key Points

**Question:**

Are preterm birth, small for gestational age, and large for gestational age associated with increased risks of atrial fibrillation (AF) up to age 49 years?

**Findings:**

In this multinational cohort study with 8 million participants, preterm birth and large for gestational age were associated with increased risks of AF in childhood and up to age 49 years in adulthood, while an association between small for gestational age and an increased risk of AF was observed only in childhood. Similar findings were observed in sibling analyses.

**Meaning:**

Preterm birth, excessive fetal growth, and reduced fetal growth may increase the risk of AF up to age 49 years.

## Teaser

This study investigates whether preterm birth, small for gestational age, or large for gestational age are associated with increased risks of atrial fibrillation later in life.

## Introduction

Atrial fibrillation (AF), the most common cardiac arrhythmia of clinical significance, is a growing global epidemic.^1^ It is associated with increased mortality^2^ and morbidity, primarily due to stroke^3^ and heart failure.^4^ Although AF primarily affects middle aged and older individuals, it also presents in children and young adults. The incidence of AF in children and young adults has increased slightly during the past decades and its estimated prevalence is 0.12% to 0.16%.^1,5,6^ Having AF in young age may entail a higher socioeconomic burden than in old age because of the lifelong work productivity loss and the increased health care costs. The well-established risk factors for AF, such as age, male sex, genetic factors, smoking, obesity, hypertension, diabetes, and specific heart diseases,^7^ do not explain a substantial proportion of AF cases in children and young adults.^8,9^ Knowledge on the etiology of AF in children and young adults is lacking, prompting the need for further studies in this area.

Restricted and excessive fetal growth, as reflected in small and large for gestational age (SGA and LGA, respectively), have been linked to increased risks of cardiovascular diseases (CVD), including hypertension,^10^ ischemic heart disease (IHD),^11^ stroke,^12^ heart failure,^13^ and other cardiometabolic disorders in childhood and adulthood.^14,15^ Short gestational age, as reflected in preterm birth, was also found to be associated with increased risks of IHD, stroke, and heart failure, irrespective of fetal growth.^16,17,18,19^ Knowledge regarding associations between fetal growth and risks of AF is limited and inconsistent^20,21,22,23,24,25^; some studies reported positive, others inverse, *U*-shaped, or no associations between birth weight and AF.^20,21,22,23,24,25^ The 2 studies regarding preterm birth and AF found no association.^22,23^ All of the earlier studies were conducted in populations that were middle aged or older at baseline. Thus, several of these studies were prone to recall bias, survival bias, or did not consider important confounders, including familial risk factors. None of them examined both the individual and the joint effects of fetal growth and gestational age or the role of these exposures in pediatric and adult AF.

In this population-based study using prospectively recorded nationwide data on more than 8 million individuals from 3 Nordic countries who were followed up to age 49 years, we investigated whether preterm birth, SGA, or LGA are associated with AF risk in childhood and adulthood. Given the strong association between preterm birth and poor fetal growth,^26^ we also examined their joint effects. To consider confounding by shared familial factors, we performed analyses with a sibling design.

## Methods

### Study Population

We conducted a cohort study by linking several nationwide registers in Denmark, Sweden, and Finland through the unique personal identification number assigned to each resident. A detailed description of the registers included and the main variables retrieved is provided in eAppendix 1 in Supplement 1. Using the medical birth registers, we identified all live singletons in Denmark from 1978 through 2016 (n = 2 332 882), in Sweden from 1973 through 2014 (n = 4 171 006), and a random sample of 90% live singletons in Finland from 1987 through 2014 (n = 1 636 116). After excluding births with missing or implausible gestational age, birth weight, or sex, our study population consisted of 8 012 433 births (eFigure 1 in Supplement 1). The study was approved by the Danish Data Protection Agency and the Research Ethics Committee in Stockholm, Sweden. The boards do not request informed consent for register-based studies. We followed the Strengthening the Reporting of Observational Studies in Epidemiology (STROBE) reporting guidelines.

### Measures

#### Exposures

Information on gestational age and birth weight was retrieved from the medical birth registers. Estimation of gestational age was based primarily on ultrasound examinations performed in the early second trimester or otherwise on the date of the last menstrual period. Preterm birth was defined as birth before 37 completed weeks of gestation and was categorized as extremely and very preterm (22 to 31 weeks) and moderately preterm (32 to 36 weeks).

We calculated birth weight for gestational age according to a Scandinavian sex-specific reference curve for normal fetal growth^27^ and categorized it as SGA (less than 10th percentile of each country’s distribution), LGA (more than 90th percentile), and appropriate for gestational age ([AGA] 10th to 90th percentile). The SGA and LGA were further divided into severe SGA (less than 3rd percentile), moderate SGA (3rd to less than 10th percentile), moderate LGA (more than 90th to 97th percentile), and severe LGA (more than 97th percentile).

#### Outcome

AF was defined as AF or atrial flutter, given their close interrelationships.^28^ We identified individuals with a primary or secondary diagnosis of AF from the Danish National Patient Register, the Swedish Patient Register, and the Finnish Hospital Discharge Register using the *International Classification of Diseases *codes shown in eTable 1 in Supplement 1. Follow-up started at birth and ended on the date of the first AF diagnosis, death, emigration, or the latest date with available data (December 31, 2016, in Denmark, December 31, 2021, in Sweden, and December 31, 2014, in Finland), whichever came first.

#### Covariates

We obtained information on children’s characteristics (ie, sex, year of birth, country of birth, BMI, cardiac surgery, diagnoses of congenital anomalies, hypertension, diabetes, IHD, stroke, and heart failure), and maternal characteristics (ie, country of origin, education, marital status, age at delivery, parity, smoking, BMI in early pregnancy, hypertensive and diabetic disorders before delivery, and family history of CVD), as described in eAppendix 2 in Supplement 1.

### Statistical Analyses

**Figure 1.**
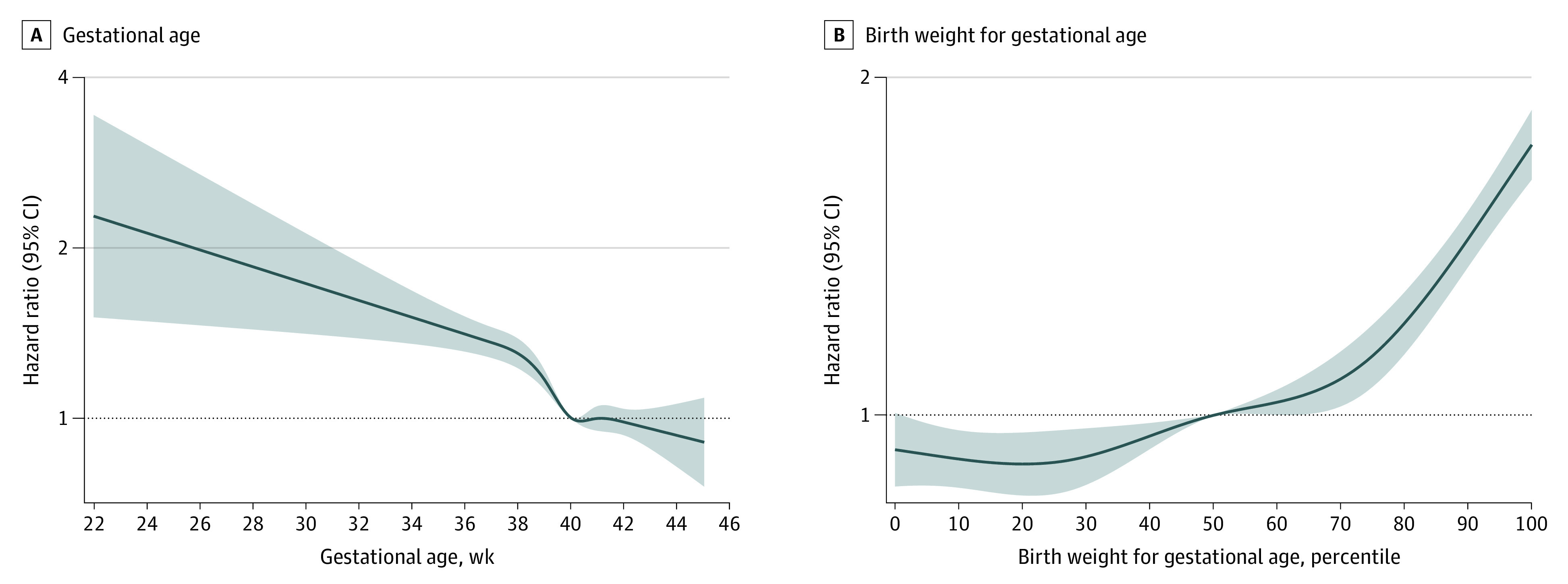
Adjusted Hazard Ratios for Atrial Fibrillation According to Gestational Age (Weeks) and Birth Weight for Gestational Age (Percentiles) We placed 5 knots at the 5th, 25th, 50th, 75th, and 95th percentiles. We adjusted for country, calendar year of birth, maternal parity, age, education, marital status, hypertensive disorders, and diabetes before childbirth. The model with gestational age was further adjusted for the child’s sex and birth weight.

We first investigated the associations between gestational age and birth weight for gestational age as continuous variables and AF risk. We applied restricted cubic spline functions with 5 knots, located at the 5th, 25th, 50th, 75th, and 95th percentiles of the distribution of each variable. Given the nonlinearity of the associations (Figure 1), we further analyzed gestational age and birth weight for gestational age as categorical variables.

We estimated hazard ratios (HRs) and 95% CIs for the associations between preterm birth, SGA, and LGA and the risk of AF by Cox proportional hazard regression using attained age as the underlying time scale. The log-minus log-survival curves and the Schoenfeld residuals suggested that the proportional hazard assumption does not hold for our exposures. To account for the nonproportional effects, we performed analyses with the follow-up time split at 18 years. We also used flexible parametric survival models^29^ to visualize the time-varying effects. In our main models, we adjusted for the study participants’ year of birth and country of birth and for maternal characteristics, ie, parity, age at delivery, education, marital status, hypertensive disorders, and diabetes before delivery. Analyses of preterm birth were also adjusted for sex and birth weight. To control for unmeasured familial confounders, we conducted sibling analyses. We ran stratified Cox models with a separate stratum for each family; only sibling pairs discordant for exposure and outcome contributed to the estimates. We also analyzed the joint effects of gestational age and birth weight for gestational age on AF risk.

To allow comparisons with earlier studies, we examined AF risk according to birth weight categorized as low (less than 2500 g), medium (2500 to 3999 g), or high (4000 g or more). To test whether the association between preterm birth or abnormal fetal growth and AF differed by the participants’ country at birth or sex, we conducted stratified analyses and formal tests of interaction with these variables. In sensitivity analyses we: (1) changed the start of follow-up to the age of 1 year to exclude the possibility that AF may be an immediate complication of treatments during neonatal care; (2) performed analyses among participants without congenital anomalies; (3) adjusted for maternal smoking or BMI in early pregnancy, maternal country of origin, or maternal family history of CVD in addition to covariates in our main model, restricting to individuals with data on these variables; (4) adjusted one by one for participants’ BMI, hypertension, diabetes, IHD, stroke, and heart failure, in addition to covariates in our main model to examine whether these factors could contribute to the observed associations; and (5) repeated analyses in the Danish subcohort after excluding individuals who underwent cardiac surgery during the follow-up period to consider that postoperative AF has a different origin than other AFs. Statistical analyses were performed using SAS version 9.4 (SAS Institute) and Stata version 15.1 (StataCorp).

## Results

A total of 378 917 of our study participants were born preterm (4.7%), 800 959 SGA (10.0%) and 802 759 LGA (10.0%). The maximum age at the end of follow-up was 49 years (median, 21 years; IQR, 11.7-30.7 years). During the 174.4 million person-years follow-up, 11 464 participants had AF (0.14%); the median age at diagnosis was 29.3 years (IQR, 22.3-36.4 years). Characteristics of the cohort according to exposures are presented in eTable 2 in Supplement 1. Compared with term births, preterm births were more likely to be firstborn or with congenital anomalies; mothers who experienced a preterm birth were more likely to smoke or have a low educational level or hypertensive disorders. Similar patterns were observed when comparing SGA with AGA births. Compared with AGA births, LGA births were more like to have mothers who were multiparous, older, obese, or had diabetes.

### Gestational Age and AF Risk

**Figure 2.**
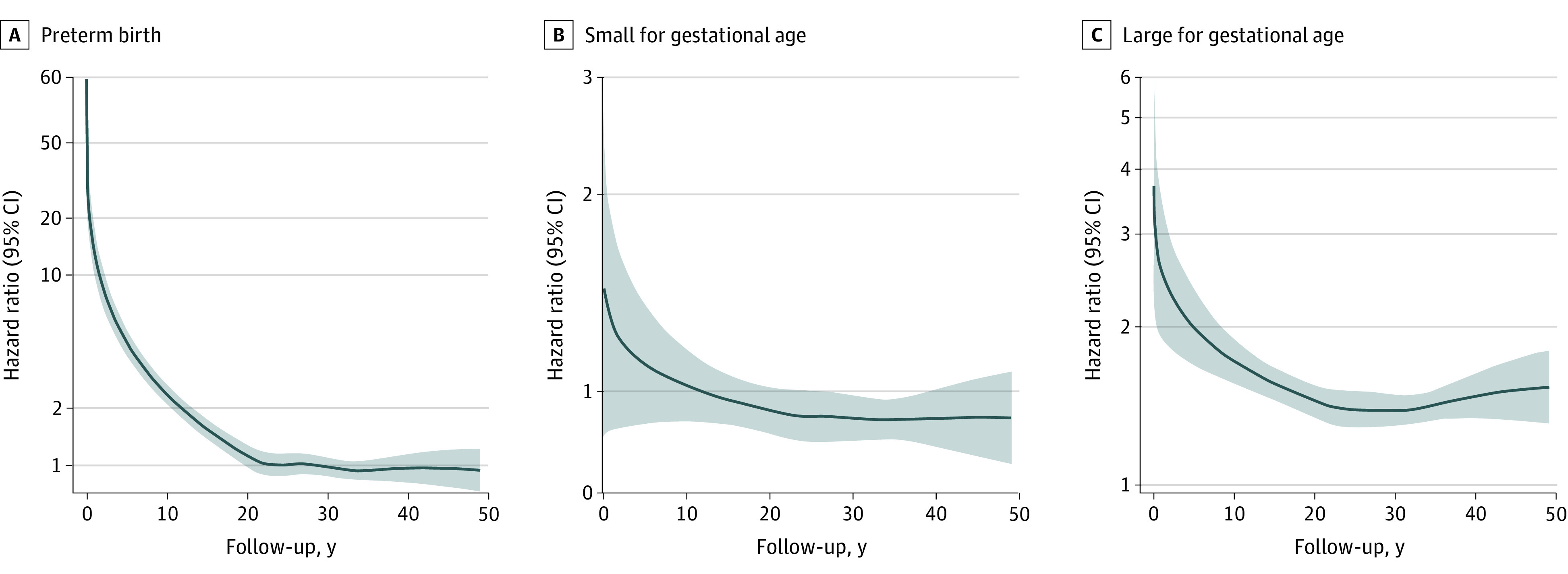
Adjusted Hazard Ratios for Atrial Fibrillation According to Preterm Birth, Small for Gestational Age, and Large for Gestational Age From Flexible Parametric Survival Models A spline with 5 *df* (4 intermediate knots and 2 knots at each boundary, placed at quintiles of the distribution of events) was used for the baseline rate and a spline with 3 *df* was used for the time-varying effect. We adjusted for country, calendar year of birth, maternal parity, age, education, marital status, hypertensive disorders, and diabetes before childbirth. The model with preterm birth was further adjusted for the child’s sex and birth weight.

The study team observed an inverse, largely linear association between gestational age and AF risk (Figure 1). Preterm birth was associated with an increased AF risk during follow-up (eFigure 2 in Supplement 1); the adjusted HRs were 1.30 (95% CI, 1.18-1.42) in the population and 1.29 (95% CI, 1.08-1.55) in the sibling analysis (Table 1). The flexible parametric survival model (Figure 2) revealed that the association attenuated over time; a similar pattern was also seen when splitting follow-up time, ie, estimates observed in the first 18 years of the follow-up had larger effect sizes than those observed afterwards (Table 1).

**Table 1.** Incidence Rates and Hazard Ratios (HRs) With 95% CIs for Atrial Fibrillation According to Preterm Birth

Exposure	Population analysis (n = 8 012 433)				Sibling analysis (n = 6 614 184)			
	No. of events	Rate, per 10 000 person-years	Crude HR (95% CI)	Adjusted HR (95% CI)^a^	No. of events	Rate, per 10 000 person-years	Crude HR (95% CI)	Adjusted HR (95% CI)^a^
**Overall follow-up**								
Term	10 815	0.65	1 [Reference]	1 [Reference]	8113	0.59	1 [Reference]	1 [Reference]
Preterm	649	0.82	1.28 (1.18-1.39)	1.30 (1.18-1.42)	472	0.76	1.40 (1.20-1.64)	1.29 (1.08-1.55)
**First 18 y of follow-up**								
Term	1218	0.11	1 [Reference]	1 [Reference]	1017	0.11	1 [Reference]	1 [Reference]
Preterm	132	0.25	2.26 (1.89-2.70)	2.36 (1.90-2.94)	98	0.23	2.21 (1.57-3.11)	2.33 (1.57-3.46)
**After 18 y of follow-up^b^**								
Term	9597	1.75	1 [Reference]	1 [Reference]	7096	1.63	1 [Reference]	1 [Reference]
Preterm	517	2.00	1.15 (1.06-1.26)	1.16 (1.05-1.29)	374	1.91	1.25 (1.05-1.48)	1.11 (1.01-1.35)
**Finer exposure categories**								
Extremely and very preterm	44	0.52	0.93 (0.69-1.25)	0.89 (0.65-1.23)	31	0.49	0.89 (0.52-1.52)	0.82 (0.45-1.48)
Moderately preterm	605	0.85	1.32 (1.21-1.43)	1.32 (1.20-1.45)	441	0.79	1.45 (1.24-1.70)	1.31 (1.10-1.57)

^a^ Adjusted for country, calendar year of birth, sex, birth weight, maternal parity, age, education, marital status, hypertensive disorders, and diabetes before childbirth.

^b^ After 18 years of follow-up, there were 4 646 035 individuals in the population analysis, and 3 877 746 in the sibling analysis.

### Birth Weight for Gestational Age and AF Risk

The risk of AF increased substantially by birth weight for gestational age from the 50th percentile (Figure 1). Compared with individuals born AGA, individuals born SGA were at lower AF risk (adjusted HR, 0.93; 95% CI, 0.88-0.99), while individuals born LGA were at higher AF risk (adjusted HR, 1.55; 95% CI, 1.46-1.63). In the sibling analysis, only LGA was associated with AF risk. In both population and sibling analyses, the AF risk was higher for severe than for moderate LGA (Table 2).

**Table 2.** Incidence Rates and Hazard Ratios (HRs) for Atrial Fibrillation According to Birth Weight for Gestational Age

Exposure	Population analysis (n = 8 012 433)				Sibling analysis (n = 6 614 184)			
	No. of events	Rate, per 10 000 person-years	Crude HR (95% CI)	Adjusted HR (95% CI)^a^	No. of events	Rate, per 10 000 person-years	Crude HR (95% CI)	Adjusted HR (95% CI)^a^
**Overall follow-up**								
SGA	1270	0.69	0.96 (0.90-1.02)	0.93 (0.88-0.99)	877	0.62	1.08 (0.96-1.21)	1.05 (0.93-1.18)
AGA	8684	0.63	1 [Reference]	1 [Reference]	6571	0.57	1 [Reference]	1 [Reference]
LGA	1510	0.88	1.54 (1.46-1.63)	1.55 (1.46-1.63)	1137	0.78	1.25 (1.13-1.39)	1.29 (1.15-1.43)
**First 18 y of follow-up**								
SGA	161	0.14	1.26 (1.07-1.49)	1.27 (1.07-1.50)	122	0.13	1.33 (0.99-1.78)	1.41 (1.04-1.91)
AGA	1005	0.11	1 [Reference]	1 [Reference]	838	0.11	1 [Reference]	1 [Reference]
LGA	184	0.15	1.44 (1.23-1.69)	1.44 (1.23-1.68)	155	0.15	1.07 (0.82-1.40)	1.02 (0.78-1.35)
**After 18 y of follow-up^b^**								
SGA	1109	1.65	0.92 (0.87-0.98)	0.90 (0.84-0.96)	755	1.51	1.04 (0.91-1.18)	0.99 (0.87-1.14)
AGA	7679	1.69	1 [Reference]	1 [Reference]	5733	1.58	1 [Reference]	1 [Reference]
LGA	1326	2.53	1.55 (1.47-1.65)	1.56 (1.47-1.66)	982	2.32	1.29 (1.15-1.45)	1.34 (1.19-1.51)
**Finer exposure categories**								
Severe SGA	426	0.75	1.02 (0.92-1.12)	0.98 (0.89-1.09)	282	0.67	1.19 (0.97-1.45)	1.16 (0.95-1.42)
Moderate SGA	855	0.66	0.93 (0.87-1.01)	0.92 (0.86-0.98)	607	0.60	1.05 (0.92-1.20)	1.03 (0.90-1.17)
AGA	8693	0.63	1 [Reference]	1 [Reference]	6572	0.57	1 [Reference]	1 [Reference]
Moderate LGA	958	0.81	1.41 (1.32-1.51)	1.42 (1.32-1.51)	732	0.72	1.19 (1.05-1.34)	1.22 (1.08-1.38)
Severe LGA	532	1.05	1.89 (1.73-2.06)	1.88 (1.73-2.06)	392	0.92	1.44 (1.22-1.70)	1.49 (1.25-1.76)

Abbreviations: AGA, appropriate for gestational age; LGA, large for gestational age; SGA, small for gestational age.

^a^ Adjusted for country, calendar year of birth, maternal parity, age, education, marital status, hypertensive disorders, and diabetes before childbirth.

^b^ After 18 years of follow-up, there were 4 646 035 individuals in the population analysis and 3 877 746 in the sibling analysis.

Being SGA was associated with an increased risk of AF during the first 18 years of follow-up (adjusted HR, 1.27; 95% CI, 1.07-1.50) but with a reduced risk later (adjusted HR, 0.90; 95% CI, 0.84-0.96); the association between LGA and AF risk was similar between the 2 periods. The flexible parametric models showed that the strength of the associations with SGA or LGA declined with increasing age at follow-up (Figure 2). Both low and high birth weight were associated with increased AF risk; after adjusting for gestational age, the association remained only in cases of high birth weight (eTable 3 in Supplement 1).

### Combination of Gestational Age and Birth Weight for Gestational Age

**Figure 3.**
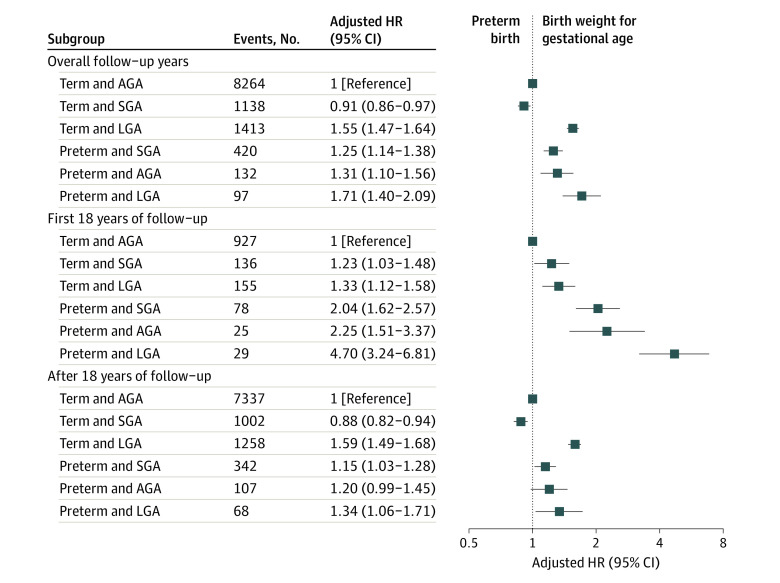
Adjusted Hazard Ratios (HRs) and 95% CIs for Atrial Fibrillation According to Preterm Birth and Birth Weight for Gestational Age Term appropriate for gestational age (AGA) births were used as the reference and analyses were adjusted for country, calendar year of birth, maternal parity, age, education, marital status, hypertensive disorders, and diabetes before childbirth. SGA indicates small for gestational age; LGA, large for gestational age.

Compared with term AGA births, the risk of AF was increased by 71% in preterm LGA births, 55% in term LGA births, 31% in preterm AGA births, and 25% in preterm SGA births. The risk of AF was 9% lower for term SGA than term AGA births (Figure 3). In these analyses, the HRs were generally higher in the first 18 years of follow-up than later.

### Sensitivity Analyses

The associations of preterm birth, SGA, and LGA with AF risk did not differ by the child’s sex or country of birth (eTable 4 and eFigure 3 in Supplement 1). The results remained essentially unchanged when the study team: (1) set the start of the follow-up to the age of 1 year (eTable 5 in Supplement 1); (2) restricted the analyses to individuals without congenital anomalies (eTable 6 in Supplement 1); (3) added maternal smoking or BMI during early pregnancy, country of origin, or family history of CVD to the main model (eTable 7 through eTable 10 in Supplement 1); (4) adjusted for the study participants’ BMI, hypertension, diabetes, IHD, stroke, and heart failure in addition to the factors in the main model (eTable 11 in Supplement 1); or (5) repeated the main analyses in the Danish subcohort after excluding individuals who had undergone cardiac surgery during the follow-up (n = 8268) (data not shown).

## Discussion

In this large population-based study, we found that preterm birth and LGA were associated with increased risks of AF up to the age of 49 years. The highest AF risk was observed in study participants who were both preterm and LGA. These associations persisted from childhood into early middle age and were also generally observed in the sibling analyses. Individuals born SGA had an increased risk of AF in childhood, both when compared with the general population and with their siblings.

Evidence regarding the link between gestational age or fetal growth and the risk of AF has been limited and inconsistent. In contrast to this study, a Swedish cohort study^22^ and the Helsinki Birth Cohort Study^23^ found no association between preterm birth and AF risk. A possible explanation for the differences in results may be related to survival bias, as the participants included in the 2 previous studies were born between 1914 and 1952, ie, when no advanced neonatal care was available. Thus, individuals who survived after a preterm birth in the early 20th century were likely to be healthier than the survivors of preterm birth from our study. The 2 earlier studies reported *U*-shaped associations between birth weight and the risk of AF after adjusting for gestational age, but the associations were generally weak. Similar to this study, the Women’s Health Study^21^ also found a link between high birth weight and an increased AF risk, but the inclusion of only healthy and well-educated women limited the generalizability of their findings. In contrast, the Atherosclerosis Risk in Communities Study^20^ found that low birth weight was associated with an increased risk of AF. These studies were limited by low statistical power, self-reported birth weight, and lack of data on several important confounders.

To our knowledge, this study is the first to investigate the association between birth outcomes and both pediatric and adult AF. Preterm birth, SGA, and LGA were associated with increased risks of pediatric AF and the associations for preterm birth and SGA had larger effect sizes than the corresponding associations with AF in young adulthood. We can only speculate about explanations for these findings. First, the etiology of pediatric AF and adult AF has been suggested to differ; children with AF are more likely to have congenital heart diseases,^30^ conditions that may be related to both preterm birth and restricted fetal growth.^31,32^ Nevertheless, when we excluded study participants with congenital anomaly, we observed similar associations to those in the main analyses. Second, an increased risk of AF in childhood could be a direct complication of or may be triggered by neonatal treatments, more commonly occurring in preterm or SGA births. To address this concern, we performed sensitivity analyses in which follow-up started at 1 year of age, but the results of these analyses were similar to those of our primary analyses.

There are several potential explanations for the link of preterm birth and abnormal fetal growth with the risk of AF. First, the associations may in part be due to shared genetic background or to maternal socioeconomic status,^33,34^ lifestyle,^35,36,37,38^ hypertensive disorders,^39,40^ and diabetes,^41,42^ or other confounders. Nevertheless, adjustment for several maternal characteristics did not change the associations considerably. The results of our sibling analyses suggest that the contributions of shared genetic and environmental factors to the associations of preterm birth and LGA with the risk of AF are likely to be modest but might be important in the case of the association between SGA and AF. Second, preterm birth, SGA, and LGA may cause cardiac remodeling and dysfunction during fetal life and/or persistent impairment of atrial structure or function during early childhood and adolescence,^43,44^ which in turn may lead to AF. Third, preterm birth, SGA, and LGA birth may lead to AF through adverse changes in cardiorespiratory fitness,^45^ blood pressure,^46,47,48^ insulin sensitivity,^49,50^ adiposity and lipid deposition,^51^ and other cardiometabolic disorders later in life.^15^ Fourth, AF occurring during cardiac surgery, potentially more common in participants with adverse birth outcomes, may also contribute to the observed associations. Nevertheless, our point estimates did not substantially change after we controlled for high BMI, diabetes, hypertension, IHD, stroke, heart failure, or cardiac surgery, suggesting that other unmeasured factors may explain the investigated associations. A potential explanation for the finding that the risk of AF was highest for the combination of preterm birth and LGA may be that maternal medical conditions, such as diabetes and/or obesity during pregnancy lead to preterm birth,^42,52^ and together induce lasting physiological alterations, such as insulin resistance, vascular endothelial dysfunction, and cardiac damage, that lead to AF.^36,41,53^

### Strengths and Limitations

The large sample size of this study allowed us to evaluate more narrowly defined gestational age and birth weight for gestational age groups, to study their associations with both pediatric and adult AF, and to perform sibling analyses to consider genetic or environmental confounders shared by siblings. The prospectively collected data from registers effectively eliminated the possibility for recall or selection bias.

Several limitations of our study should be noted. First, although the diagnoses of AF in the Nordic patient registers has a high positive predictive value,^54,55^ we may have missed some asymptomatic, paroxysmal, or mild cases of AF. Second, we cannot rule out the possibility of residual confounding. Our sibling analyses could partly control for unmeasured familial risk factors, but this design itself has several limitations like the bias caused by the carry over effects^56^ or nonshared confounders.^57^ Third, because of the limited follow-up time, our findings apply only to children and young adults and cannot be generalized to the predominant group of individuals developing AF at an older age. Additionally, our results may only be generalized to countries with predominantly White populations and universal free health care systems.

## Conclusions

In this study, preterm birth and LGA birth were associated with increased AF risks up to the age of 49 years. Individuals born SGA have an increased AF risk only in childhood. Further studies with longer follow-up and that may elucidate the underlying mechanisms for the observed associations are warranted. As the prevalence of LGA births is reported to increase over time,^58^ the possible long-term health effects of being born LGA may become increasingly important.

## Notes

Supplement 1. eTable 1. International Classification of Diseases codes for atrial fibrillation eTable 2. Baseline characteristics of the study population according to gestational age and birth weight for gestational age eTable 3. Incidence rates and hazard ratios with 95% confidence intervals for atrial fibrillation according to birth weight eTable 4. Incidence rates and hazard ratios with 95% confidence intervals for atrial fibrillation according to preterm birth and birth weight for gestational age, stratified by sex eTable 5. Incidence rates and hazard ratios with 95% confidence intervals for atrial fibrillation according to preterm birth and birth weight for gestational age, when follow-up started at the age of one year eTable 6. Incidence rates and hazard ratios with 95% confidence intervals for atrial fibrillation according to preterm birth and birth weight for gestational age, in study participants without congenital anomalies eTable 7. Incidence rates and hazard ratios with 95% confidence intervals for atrial fibrillation according to preterm birth and birth weight for gestational age, in births with information on maternal smoking during early pregnancy eTable 8. Incidence rates and hazard ratios with 95% confidence intervals for atrial fibrillation according to preterm birth and birth weight for gestational age, in births with information on maternal body-mass index during early pregnancy eTable 9. Incidence rates and hazard ratios with 95% confidence intervals for atrial fibrillation according to preterm birth and birth weight for gestational age, in births with information on maternal family history of cardiovascular diseases eTable 10. Incidence rates and hazard ratios with 95% confidence intervals for atrial fibrillation according to preterm birth and birth weight for gestational age, in births with information on maternal country of origin eTable 11. Incidence rates and hazard ratios with 95% confidence intervals for atrial fibrillation according to preterm birth and birth weight for gestational age, after adjusting for the study participants’ cardiometabolic diseases eFigure 1. Flowchart of the study population eFigure 2. Cumulative incidence of atrial fibrillation according to preterm birth and birth weight for gestational age eFigure 3. Adjusted hazard ratios and 95% confidence intervals for atrial fibrillation according to preterm birth and birth weight for gestational age, stratified by study country eAppendix 1. Description of the registers used in the study eAppendix 2. Description of the source, the measurement and the categorization of covariates eReferences

Supplement 2. Data sharing statement
