# Supplementary material for: Preterm Birth, Small for Gestational Age, and Large for Gestational Age and the Risk of Atrial Fibrillation Up to Middle Age
Source: JAMA Pediatr. 2023 Apr 24;177(6):599–607. doi: 10.1001/jamapediatrics.2023.0083 (PMC10126943; doi:10.1001/jamapediatrics.2023.0083)
Supplement: Supplement 1. — eTable 1. International Classification of Diseases codes for atrial fibrillation eTable 2. Baseline characteristics of the study population according to gestational age and birth weight for gestational age eTable 3. Incidence rates and hazard ratios with 95% confidence intervals for atrial fibrillation according to birth weight eTable 4. Incidence rates and hazard ratios with 95% confidence intervals for atrial fibrillation according to preterm birth and birth weight for gestational age, stratified by sex eTable 5. Incidence rates and hazard ratios with 95% confidence intervals for atrial fibrillation according to preterm birth and birth weight for gestational age, when follow-up started at the age of one year eTable 6. Incidence rates and hazard ratios with 95% confidence intervals for atrial fibrillation according to preterm birth and birth weight for gestational age, in study participants without congenital anomalies eTable 7. Incidence rates and hazard ratios with 95% confidence intervals for atrial fibrillation according to preterm birth and birth weight for gestational age, in births with information on maternal smoking during early pregnancy eTable 8. Incidence rates and hazard ratios with 95% confidence intervals for atrial fibrillation according to preterm birth and birth weight for gestational age, in births with information on maternal body-mass index during early pregnancy eTable 9. Incidence rates and hazard ratios with 95% confidence intervals for atrial fibrillation according to preterm birth and birth weight for gestational age, in births with information on maternal family history of cardiovascular diseases eTable 10. Incidence rates and hazard ratios with 95% confidence intervals for atrial fibrillation according to preterm birth and birth weight for gestational age, in births with information on maternal country of origin eTable 11. Incidence rates and hazard ratios with 95% confidence intervals for atrial fibrillation according to preterm birth a [file jamapediatr-e230083-s001.pdf]

Supplemental Online Content

Yang F, Janszky I, Gissler M, et al. Preterm birth, small for gestational age, and large for gestational age and the risk of atrial fibrillation up to middle age. *JAMA Pediatr*. Published online April 24, 2023. doi:10.1001/jamapediatrics.2023.0083

eTable 1. International Classification of Diseases codes for atrial fibrillation

eTable 2. Baseline characteristics of the study population according to gestational age and birth weight for gestational age

eTable 3. Incidence rates and hazard ratios with 95% confidence intervals for atrial fibrillation according to birth weight

eTable 4. Incidence rates and hazard ratios with 95% confidence intervals for atrial fibrillation according to preterm birth and birth weight for gestational age, stratified by sex

eTable 5. Incidence rates and hazard ratios with 95% confidence intervals for atrial fibrillation according to preterm birth and birth weight for gestational age, when follow-up started at the age of one year

eTable 6. Incidence rates and hazard ratios with 95% confidence intervals for atrial fibrillation according to preterm birth and birth weight for gestational age, in study participants without congenital anomalies

eTable 7. Incidence rates and hazard ratios with 95% confidence intervals for atrial fibrillation according to preterm birth and birth weight for gestational age, in births with information on maternal smoking during early pregnancy

eTable 8. Incidence rates and hazard ratios with 95% confidence intervals for atrial fibrillation according to preterm birth and birth weight for gestational age, in births with information on maternal body-mass index during early pregnancy

eTable 9. Incidence rates and hazard ratios with 95% confidence intervals for atrial fibrillation according to preterm birth and birth weight for gestational age, in births with information on maternal family history of cardiovascular diseases

eTable 10. Incidence rates and hazard ratios with 95% confidence intervals for atrial fibrillation according to preterm birth and birth weight for gestational age, in births with information on maternal country of origin

eTable 11. Incidence rates and hazard ratios with 95% confidence intervals for atrial fibrillation according to preterm birth and birth weight for gestational age, after adjusting for the study participants' cardiometabolic diseases

eFigure 1. Flowchart of the study population

eFigure 2. Cumulative incidence of atrial fibrillation according to preterm birth and birth weight for gestational age

eFigure 3. Adjusted hazard ratios and 95% confidence intervals for atrial fibrillation according to preterm birth and birth weight for gestational age, stratified by study country

© 2023 Yang F et al. *JAMA Pediatrics*.

eAppendix 1. Description of the registers used in the study

eAppendix 2. Description of the source, the measurement and the categorization of covariates

eReferences

This supplemental material has been provided by the authors to give readers additional information about their work.

© 2023 Yang F et al. *JAMA Pediatrics*.

eTable 1. International Classification of Diseases codes for atrial fibrillation

	ICD-8	ICD-9	ICD-10
Denmark [a]	427.93, 427.94		I48
Sweden [b]	427.92	427D	I48
Finland [c]		4273	I48

Abbreviations: ICD, International Statistical Classification of Diseases and Related Health Problems.
[a] In Denmark, information on atrial fibrillation was extracted from the Danish National Patient Register. During the study period, Denmark used ICD-8 until 1995 and ICD-10 afterwards.
[b] In Sweden, information on atrial fibrillation was extracted from the Swedish Patient Register. During the study period, Sweden used ICD-8 until 1986, ICD-9 from 1987 to 1996, and ICD-10 afterwards.
[c] In Finland, information on atrial fibrillation was extracted from the Finnish Hospital Discharge Register. During the study period, Finland used ICD-9 until 1995 and ICD-10 afterwards.

© 2023 Yang F et al. *JAMA Pediatrics*.

eTable 2. Baseline characteristics of the study population according to gestational age and birth weight for gestational age

Variables	Gestational age		Birth weight for gestational age		
	Term birth (N=7,633,516)	Preterm birth (N=378,917)	AGA (N=6,408,715)	SGA (N=800,959)	LGA (N=802,759)
	No. (%)	No. (%)	No. (%)	No. (%)	No. (%)
**Children's characteristics**					
**Calendar year of birth**					
1973-1978	604,264 (7.92)	28,060 (7.41)	485,402 (7.57)	100,525 (12.55)	46,397 (5.78)
1979-1984	784,538 (10.28)	39,479 (10.42)	655,910 (10.23)	96,750 (12.08)	71,357 (8.89)
1985-1990	1,153,731 (15.11)	59,494 (15.70)	969,107 (15.12)	123,441 (15.41)	120,677 (15.03)
1991-1996	1,338,468 (17.53)	65,515 (17.29)	1,119,704 (17.47)	126,043 (15.74)	158,236 (19.71)
1997-2002	1,146,900 (15.02)	57,442 (15.16)	962,690 (15.02)	108,146 (13.50)	133,514 (16.63)
2003-2008	1,230,015 (16.11)	62,241 (16.43)	1,043,372 (16.28)	114,346 (14.28)	134,538 (16.76)
2009-2016	1,375,592 (18.02)	66,686 (17.60)	1,172,530 (18.30)	131,708 (16.44)	138,040 (17.20)
**Sex**					
Male	3,904,467 (51.15)	208,729 (55.09)	3,288,406 (51.31)	399,152 (49.83)	425,638 (53.02)
Female	3,729,049 (48.85)	170,188 (44.91)	3,120,309 (48.69)	401,807 (50.17)	377,121 (46.98)
**Congenital anomalies**					
No	6,966,474 (91.26)	319,549 (84.33)	5,85,837 (91.30)	706,771(88.24)	728,415 (90.74)
Yes	667,042 (8.74)	59,368 (15.67)	557,878 (8.70)	94,188 (11.76)	74,344 (9.26)
**Body-mass index during follow-up** [a]					
<18.5	33,404 (2.86)	1,391 (2.98)	26,630 (2.78)	6,815 (4.17)	1,350 (1.44)
18.5–24.9	625,714 (53.61)	23,962 (51.28)	517,388 (54.07)	86,625 (53.01)	45,663 (48.84)
25.0–29.9	271,581 (23.27)	11,211 (23.99)	222,172 (23.22)	35,929 (21.99)	24,691 (26.41)
≥30.0	138,917 (11.90)	6,110 (13.08)	111,995 (11.70)	18,548 (11.35)	14,484 (15.49)
Unknown	97,475 (8.35)	4,055 (8.68)	78,715 (8.23)	15,502 (9.49)	7,313 (7.82)
**Diabetes during follow-up**					
No	7,581,072 (99.31)	375,736 (99.16)	6,365,690 (99.33)	794,735 (99.22)	796,383 (99.21)
Yes	52,444 (0.69)	3,181 (0.84)	43,025 (0.67)	6,224 (0.78)	6,376 (0.79)
**Hypertension during follow-up**					
No	7,591,331 (99.45)	375,947 (99.22)	6,374,582 (99.47)	793,524 (99.07)	799,172 (99.55)
Yes	42,185 (0.55)	2,970 (0.78)	34,133 (0.53)	7,435 (0.93)	3,587 (0.45)
**Ischemic heart disease during follow-up**					
No	7,627, 677 (99.92)	378,542 (99.90)	6,404,067 (99.93)	799,886 (99.87)	802,266 (99.49)

© 2023 Yang F et al. *JAMA Pediatrics.*

Yes	5,839 (0.08)	375 (0.10)	4,648 (0.07)	1,073 (0.13)	493 (0.06)
**Stroke during follow-up**					
No	7,623,059 (99.86)	378,213 (99.81)	6,400,221 (99.87)	799,321 (99.80)	801,730 (99.87)
Yes	10,457 (0.14)	704 (0.19)	8,494 (0.13)	1,638 (0.20)	1,029 (0.13)
**Heart failure during follow-up**					
No	7,630,058 (99.95)	378,618 (99.92)	6,405,957 (99.96)	800,253 (99.91)	802,466 (99.96)
Yes	3,458 (0.05)	299 (0.08)	2,758 (0.04)	706 (0.09)	293 (0.04)
**Cardiac surgery during follow-up[b]**					
No	2,124,982 (99.66)	104,835 (99.04)	1,783,209 (99.67)	222,111 (99.25)	224,497 (99.70)
Yes	7,248 (0.34)	1,020 (0.96)	5,898 (0.33)	1,686 (0.75)	684 (0.30)
**Maternal characteristics**					
**Country of origin same as the study country [c]**					
No	730,179 (12.01)	38,385 (12.45)	616,773 (12.07)	93,431 (14.64)	58,258 (9.10)
Yes	5,344,847 (87.93)	269,663(87.48)	4,488,556 (87.87)	544,262 (85.26)	581,692 (90.87)
Unknown	3,464 (0.06)	206 (0.07)	2,807 (0.05)	650 (0.10)	213 (0.03)
**Age at the child's birth (years)**					
≤19	206,401 (2.70)	14,339 (3.78)	173,325 (2.70)	34,544 (4.31)	12,871 (1.60)
20-24	1,423,674 (18.65)	74,170 (19.57)	1,201,282 (18.74)	184,512 (23.04)	112,050 (13.96)
25-29	2,678,372 (35.09)	124,053 (32.74)	2,263,476 (35.32)	273,034 (34.09)	265,519 (33.13)
30-34	2,225,953 (29.16)	102,884(27.15)	1,862,822 (29.07)	203,114 (25.36)	262,901 (32.75)
≥35	1,099,081 (14.40)	63,468 (16.75)	907,779 (14.16)	105,752 (13.20)	149,018 (18.56)
Unknown	35(<0.01)	3 (<0.01)	31 (<0.01)	3 (<0.01)	4 (<0.01)
**Level of education before delivery**					
Primary and lower secondary	1,246,300 (16.33)	75,275 (19.87)	1,030,948 (16.09)	174,850 (21.83)	115,777 (14.42)
Upper secondary	3,757,046 (49.22)	185,605 (48.98)	3,150,394 (49.16)	382,126 (47.71)	410,131 (51.09)
Bachelor or higher	2,335,244 (30.59)	101,337 (26.74)	1,983,791 (30.95)	202,668 (25.30)	250,122 (31.16)
Unknown	294,926 (3.86)	16,700 (4.41)	243,582 (3.80)	41,315 (5.16)	26,729 (3.33)
**Marital status before delivery**					
Not married/registered partnership	3,387,418 (44.38)	185,551 (48.97)	2,863,293 (44.68)	383,330 (47.86)	326,346 (40.65)
Married/registered partnership	4,054,720 (53.12)	184,331 (48.65)	3,388,852 (52.88)	390,883 (48.80)	459,316 (57.22)
Unknown	191,378 (2.51)	9,035 (2.38)	156,570(2.44)	26,746 (3.34)	17,097 (2.13)
**Parity**					
1	3,575,422 (46.84)	203,271 (53.65)	3,042,072 (47.47)	478,477 (59.74)	258,144 (32.16)
2	2,552,938 (33.44)	101,198 (26.71)	2,133,795 (33.30)	202,729 (25.31)	317,612 39.57)

© 2023 Yang F et al. *JAMA Pediatrics.*

≥3	1,504,450 (19.71)	74,361 (19.62)	1,232,252(19.23)	119,625 (14.94)	226,934 (28.27)
Unknown	706 (0.01)	87 (0.02)	596 (0.01)	128 (0.02)	69 (0.01)
**Smoking in early pregnancy**					
No	4,799,372 (62.87)	221,553 (58.47)	4,059,876 (63.35)	396,640 (49.52)	564,409 (70.31)
Yes	1,114,575 (14.60)	67,911 (17.92)	928,994 (14.50)	167,245 (20.88)	86,247 (10.74)
Unknown	1,719,569 (22.53)	89,453 (23.61)	1,419,845 (22.15)	237,074 (29.60)	152,103 (18.95)
**Body-mass index in early pregnancy (kg/m^2) [c]**					
<18.5	120,892 (1.99)	7,927 (2.57)	103,318 (2.02)	20,504 (3.21)	4997 (0.78)
18.5–24.9	1,931,329 (31.77)	89,339 (28.98)	1,670,618 (32.71)	182,448 (28.58)	167,602 (26.18)
25.0–29.9	741,609 (12.20)	35,713 (11.59)	615,589 (12.05)	54,923 (8.60)	106,810 (16.68)
≥30.0	314,370 (5.17)	18,458 (5.99)	248,143(4.86)	23,295 (3.65)	61,390 (9.59)
Unknown	2,970,188 (48.86)	156,817 (50.87)	2,470,468 (48.36)	357,173 (48.87)	299,364 (46.76)
**Diabetes before childbirth**					
No	7,438,734 (97.45)	361,323 (95.36)	6,261,332 (97.70)	787,361 (98.30)	751,362 (93.60)
Yes	194,784 (2.55)	17,594 (4.64)	147,383 (2.30)	13,598 (1.70)	51,397(6.40)
**Hypertensive disorders before childbirth**					
No	7,360,874 (96.43)	332,863 (87.85)	6,189,749 (96.58)	733,460 (91.57)	770,541 (95.98)
Yes	272,669 (3.57)	46,054 (12.15)	218,966 (3.42)	67,499 (8.43)	32,258 (4.02)
**Family history of cardiovascular disease [c]**					
No	4,721,177 (77.67)	235,924 (76.54)	3,969,360 (77.71)	503,836 (78.93)	483,905 (75.59)
Yes	1,357,211 (22.33)	72,330 (23.46)	1,138,776 (22.29)	134,507 (21.07)	156,258 (24.41)

Abbreviations: SGA, small for gestational age; AGA, appropriate for gestational age; LGA, large for gestational age.
[a] Available only for women who later became pregnant in Sweden and Denmark (N=1,112,290).
[b] Available only in Denmark.
[c] Available only in Sweden and Denmark.

© 2023 Yang F et al. *JAMA Pediatrics.*

eTable 3. Incidence rates and hazard ratios with 95% confidence intervals for atrial fibrillation according to birth weight

Exposure	Number of events	Rate, per 10,000 person-years	Crude HR (95% CI)	Adjusted HR (95% CI) [a]
**Overall follow-up**				
Low birth weight	423	0.74	1.21 (1.10-1.34)	0.97 (0.87-1.09)
Medium birth weight	8,496	0.61	1.00	1.00
High birth weight	2,545	0.86	1.52 (1.46-1.59)	1.44 (1.37-1.51)
**First 18 years of follow-up (N=8,012,433)**				
Low birth weight	69	0.18	1.63 (1.28-2.08)	0.81 (0.59-1.11)
Medium birth weight	1028	0.11	1.00	1.00
High birth weight	253	0.12	1.13 (0.98-1.30)	1.21 (1.05-1.40)
**After 18 years of follow-up (N=4,646,035)**				
Low birth weight	354	1.84	1.16 (1.04-1.29)	0.99 (0.87-1.11)
Medium birth weight	7,468	1.61	1.00	1.00
High birth weight	2,292	2.50	1.58 (1.51-1.66)	1.47 (1.40-1.54)

Abbreviations: HR, hazard ratio; CI, confidence interval.

[a] We adjusted for country, calendar year of birth, gestational age, sex, maternal parity, age, education, marital status, hypertensive disorders and diabetes before childbirth.

© 2023 Yang F et al. *JAMA Pediatrics*.

eTable 4. Incidence rates and hazard ratios with 95% confidence intervals for atrial fibrillation according to preterm birth and birth weight for gestational age, stratified by sex [c]

Exposure	Boy (N=4,113,196)				Girl (N=3,899,237)			
	Number of events	Rate, per 10,000 person-years	Crude HR (95% CI)	Adjusted HR (95% CI) [a]	Number of events	Rate, per 10,000 person-years	Crude HR (95% CI)	Adjusted HR (95% CI) [a]
**Preterm birth**								
**Overall follow-up**								
Term	7,974	0.94	1.00	1.00	2,841	0.35	1.00	1.00
Preterm	480	1.10	1.20 (1.09-1.31)	1.29 (1.16-1.44)	169	0.47	1.38 (1.18-1.61)	1.33 (1.10-1.60)
**First 18 years of follow-up**								
Term	769	0.13	1.00	1.00	449	0.08	1.00	1.00
Preterm	84	0.28	2.11 (1.68-2.65)	2.21 (1.68-2.91)	48	0.20	2.41 (1.79-3.25)	2.67 (1.85-3.85)
**After 18 years of follow-up [b]**								
Term	7,205	2.57	1.00	1.00	2,392	0.90	1.00	1.00
Preterm	396	2.77	1.21 (1.07-1.37)	1.19 (1.05-1.34)	121	1.04	1.18 (0.98-1.41)	1.10 (0.89-1.37)
**Birth weight for gestational age**								
**Overall follow-up**								
SGA	888	0.97	0.93 (0.87-1.01)	0.92 (0.85-0.98)	382	0.41	1.07 (0.96-1.20)	1.03 (0.93-1.15)
AGA	6,384	0.90	1.00	1.00	2,300	0.34	1.00	1.00
LGA	1,182	1.29	1.09 (0.99-1.21)	1.57 (1.47-1.67)	328	0.41	1.31 (1.17-1.47)	1.34 (1.19-1.50)
**First 18 years of follow-up**								
SGA	97	0.50	1.23 (0.99-1.53)	1.25 (1.01-1.55)	64	0.11	1.33 (1.01-1.72)	1.33 (1.01-1.73)
AGA	635	0.13	1.00	1.00 (ref)	370	0.08	1.00	1.00
LGA	121	0.20	1.45 (1.19-1.76)	1.44 (1.18-1.75)	63	0.11	1.40 (1.07-1.82)	1.41 (1.07-1.84)
**After 18 years of follow-up [b]**								
SGA	791	2.36	0.91 (0.84-0.98)	0.89 (0.82-0.95)	318	0.95	1.03 (0.92-1.17)	0.99 (0.88-1.12)
AGA	5,749	2.46	1.00	1.00	1,930	0.87	1.00	1.00
LGA	1,061	3.76	1.58 (1.48-1.69)	1.58 (1.48-1.69)	265	1.09	1.30 (1.14-1.47)	1.32 (1.16-1.51)

Abbreviations: HR, hazard ratio; CI, confidence interval; SGA, small for gestational age; AGA, appropriate for gestational age; LGA, large for gestational age.
[a] We adjusted for country, calendar year of birth, maternal parity, age, education, marital status, hypertensive disorders and diabetes before childbirth. The model with preterm birth was further adjusted for the child's sex and birth weight.
[b] After 18 years of follow-up, there were 2,382,764 boys, and 2,262,271 girls included in the analyses.
[c] The p-values corresponding to the multiplicative interactions between exposure and sex were 0.16 in case of preterm birth, 0.04 in case of SGA, and 0.01 in case of LGA.

© 2023 Yang F et al. *JAMA Pediatrics.*

eTable 5. Incidence rates and hazard ratios with 95% confidence intervals for atrial fibrillation according to preterm birth and birth weight for gestational age, when follow-up started at the age of one year (N=7,853,633)

Exposure	Number of events	Rate, per 10,000 person-years	Crude HR (95% CI)	Adjusted HR (95% CI) [a]
**Preterm birth**				
**Overall follow-up**				
Term	10,596	0.64	1.00	1.00
Preterm	589	0.74	1.19 (1.09-1.29)	1.29 (1.17-1.42)
**First 17 years of follow-up (N=7,853,633)**				
Term	999	0.09	1.00	1.00
Preterm	72	0.13	1.64 (1.24-2.17)	1.50 (1.06-2.13)
**After 17 years of follow-up (N=4,646,035)**				
Term	9,597	1.75	1.00	1.00
Preterm	517	2.00	1.15 (1.06-1.26)	1.16 (1.05-1.29)
**Birth weight for gestational age**				
**Overall follow-up**				
SGA	1,250	0.68	0.96 (0.90-1.02)	0.93 (0.88-0.99)
AGA	8,486	0.61	1.00	1.00
LGA	1,449	0.85	1.52 (1.43-1.60)	1.52 (1.44-1.61)
**First 17 years of follow-up (N=7,853,633)**				
SGA	141	0.12	1.37 (1.14-1.63)	1.36 (1.13-1.63)
AGA	807	0.09	1.00	1.00
LGA	123	0.10	1.20 (0.99-1.45)	1.18 (0.98-1.43)
**After 17 years of follow-up (N=4,646,035)**				
SGA	1,109	1.65	0.92 (0.87-0.98)	0.90 (0.84-0.96)
AGA	7,679	1.69	1.00	1.00
LGA	1,326	2.53	1.55 (1.47-1.65)	1.56 (1.47-1.66)

Abbreviations: HR, hazard ratio; CI, confidence interval; SGA, small for gestational age; AGA, appropriate for gestational age; LGA, large for gestational age.

[a] We adjusted for country, calendar year of birth, maternal parity, age, education, marital status, hypertensive disorders and diabetes before childbirth. The model with preterm birth was further adjusted for the child's sex and birth weight.

© 2023 Yang F et al. *JAMA Pediatrics*.

eTable 6. Incidence rates and hazard ratios with 95% confidence intervals for atrial fibrillation according to preterm birth and birth weight for gestational age, in study participants without congenital anomalies (N=7,286,023)

Exposure	Number of events	Rate, per 10,000 person-years	Crude HR (95% CI)	Adjusted HR (95% CI) [a]
**Preterm birth**				
**Overall follow-up**				
Term	8,952	0.59	1.00	1.00
Preterm	463	0.68	1.17 (1.06-1.28)	1.21 (1.09-1.35)
**First 18 years of follow-up (N=7,286,023)**				
Term	769	0.08	1.00	1.00
Preterm	77	0.17	2.24 (1.77-2.83)	2.78 (2.12-3.64)
**After 18 years of follow-up (N=4,252,083)**				
Term	8,183	1.61	1.00	1.00
Preterm	386	1.70	1.07 (1.01-1.18)	1.08 (1.01-1.21)
**Birth weight for gestational age**				
**Overall follow-up**				
SGA	964	0.59	0.89 (0.83-0.96)	0.87 (0.81-0.93)
AGA	7,185	0.56	1.00	1.00
LGA	1,266	0.81	1.57 (1.48-1.67)	1.59 (1.49-1.68)
**First 18 years of follow-up (N=7,286,023)**				
SGA	74	0.07	0.92 (0.73-1.18)	0.92 (0.72-1.17)
AGA	647	0.08	1.00	1.00
LGA	125	0.12	1.53 (1.26-1.85)	1.54 (1.27-1.87)
**After 18 years of follow-up (N=4,252,083)**				
SGA	890	1.47	0.89 (0.83-0.95)	0.87 (0.81-0.93)
AGA	6,538	1.55	1.00	1.00
LGA	1,141	2.35	1.58 (1.48-1.68)	1.59 (1.49-1.69)

Abbreviations: HR, hazard ratio; CI, confidence interval; SGA, small for gestational age; AGA, appropriate for gestational age; LGA, large for gestational age.

[a] We adjusted for country, calendar year of birth, maternal parity, age, education level, marital status, hypertensive disorders and diabetes before childbirth. The model with preterm birth was further adjusted for the child's sex and birth weight.

© 2023 Yang F et al. *JAMA Pediatrics*.

eTable 7. Incidence rates and hazard ratios with 95% confidence intervals for atrial fibrillation according to preterm birth and birth weight for gestational age, in births with information on maternal smoking during early pregnancy (N=6,203,411)

Exposure	Number of events	Rate, per 10,000 person-years	Crude HR (95% CI)	Adjusted HR (95% CI) [a]
**Preterm birth**				
**Overall follow-up**				
Term	3,883	0.37	1.00	1.00
Preterm	9128	0.57	1.50 (1.33-1.69)	1.53 (1.33-1.77)
**First 18 years of follow-up (N=6,203,411)**				
Term	897	0.11	1.00	1.00
Preterm	94	0.24	2.17 (1.76-2.69)	2.23 (1.72-1.89)
**After 18 years of follow-up (N=2,975,903)**				
Term	2,986	1.24	1.00	1.00
Preterm	197	1.63	1.30 (1.13-1.51)	1.33 (1.12-1.58)
**Birth weight for gestational age**				
**Overall follow-up**				
SGA	381	0.38	1.05 (0.95-1.17)	1.01 (0.91-1.13)
AGA	3,142	0.35	1.00	1.00
LGA	651	0.55	1.57 (1.44-1.71)	1.58 (1.45-1.72)
**First 18 years of follow-up (N=6,203,411)**				
SGA	107	0.14	1.28 (1.04-1.56)	1.27 (1.03-1.56)
AGA	749	0.11	1.00	1.00
LGA	135	0.15	1.34 (1.11-1.60)	1.34 (1.11-1.61)
**After 18 years of follow-up (N=2,975,903)**				
SGA	274	1.17	0.99 (0.87-1.12)	0.94 (0.83-1.07)
AGA	2,393	1.18	1.00	1.00
LGA	516	1.93	1.65 (1.50-1.81)	1.66 (1.51-1.83)

Abbreviations: HR, hazard ratio; CI, confidence interval; SGA, small for gestational age; AGA, appropriate for gestational age; LGA, large for gestational age.

[a] We adjusted for country, calendar year of birth, maternal parity, age, education, marital status, hypertensive disorders and diabetes before childbirth, and maternal smoking during early pregnancy. The model with preterm birth was further adjusted for the child's sex and birth weight.

© 2023 Yang F et al. *JAMA Pediatrics*.

eTable 8. Incidence rates and hazard ratios with 95% confidence intervals for atrial fibrillation according to preterm birth and birth weight for gestational age, in births with information on maternal body-mass index during early pregnancy (N=3,259,637)

Exposure	Number of events	Rate, per 10,000 person-years	Crude HR (95% CI)	Adjusted HR (95% CI) [a]
**Preterm birth**				
**Overall follow-up**				
Term	2,107	0.38	1.00	1.00
Preterm	149	0.57	1.52 (1.29-1.79)	1.50 (1.23-1.82)
**First 18 years of follow-up (N=3,259,637)**				
Term	376	0.09	1.00	1.00
Preterm	49	0.25	2.74 (2.03-3.69)	2.29 (1.94-4.03)
**After 18 years of follow-up (N=1,429,494)**				
Term	1,731	1.24	1.00	1.00
Preterm	100	1.55	1.25 (1.02-1.53)	1.23 (0.97-1.55)
**Birth weight for gestational age**				
**Overall follow-up**				
SGA	202	0.40	1.05 (0.91-1.21)	1.01 (0.87-1.17)
AGA	1,701	0.36	1.00	1.00
LGA	353	0.57	1.62 (1.44-1.81)	1.61 (1.43-1.81)
**First 18 years of follow-up (N=3,259,637)**				
SGA	47	0.13	1.41 (1.04-1.92)	1.39 (1.02-1.89)
AGA	311	0.09	1.00	1.00
LGA	67	0.14	1.63 (1.25-2.13)	1.63 (1.24-2.13)
**After 18 years of follow-up (N=1,429,494)**				
SGA	155	1.16	0.97 (0.82-1.15)	0.94 (0.79-1.11)
AGA	1,390	1.19	1.00	1.00
LGA	286	1.89	1.61 (1.42-1.83)	1.60 (1.41-1.82)

Abbreviations: HR, hazard ratio; CI, confidence interval; SGA, small for gestational age; AGA, appropriate for gestational age; LGA, large for gestational age.

[a] We adjusted for country, calendar year of birth, maternal parity, age, education, marital status, hypertensive disorders and diabetes before childbirth, and maternal body-mass index during early pregnancy. The model with preterm birth was further adjusted for the child’s sex and birth weight.

© 2023 Yang F et al. *JAMA Pediatrics*.

eTable 9. Incidence rates and hazard ratios with 95% confidence intervals for atrial fibrillation according to preterm birth and birth weight for gestational age, in births with information on maternal family history of cardiovascular diseases (N=6,386,642)

Exposure	Number of events	Rate, per 10,000 person-years	Crude HR (95% CI)	Adjusted HR (95% CI) [a]
**Preterm birth**				
**Overall follow-up**				
Term	10,080	0.70	1.00	1.00
Preterm	596	0.86	1.26 (1.16-1.37)	1.26 (1.14-1.39)
**First 18 years of follow-up (N=6,386,642)**				
Term	957	0.10	1.00	1.00
Preterm	110	0.24	2.36 (1.94-2.88)	2.44 (1.92-3.11)
**After 18 years of follow-up (N=4,032,420)**				
Term	9,123	1.76	1.00	1.00
Preterm	486	1.98	1.14 (1.04-1.25)	1.13 (1.02-1.26)
**Birth weight for gestational age**				
**Overall follow-up**				
SGA	1,206	0.74	0.96 (0.91-1.02)	0.93 (0.88-0.99)
AGA	8,093	0.67	1.00	1.00
LGA	1,377	0.93	1.53 (1.44-1.62)	1.54 (1.45-1.63)
**First 18 years of follow-up (N=6,386,642)**				
SGA	138	0.14	1.37 (1.14-1.64)	1.36 (1.14-1.64)
AGA	786	0.10	1.00	1.00
LGA	143	0.15	1.45 (1.21-1.73)	1.45 (1.291-1.73)
**After 18 years of follow-up (N=4,032,420)**				
SGA	1,068	1.66	0.93 (0.87-0.99)	0.89 (0.84-0.96)
AGA	7,307	1.70	1.00	1.00
LGA	1,234	2.52	1.54 (1.45-1.63)	1.55 (1.46-1.65)

Abbreviations: HR, hazard ratio; CI, confidence interval; SGA, small for gestational age; AGA, appropriate for gestational age; LGA, large for gestational age.

[a] We adjusted for country, calendar year of birth, maternal parity, age, education level, marital status, family history of cardiovascular diseases, hypertensive disorders and diabetes before childbirth. The model with preterm birth was further adjusted for the child's sex and birth weight.

© 2023 Yang F et al. *JAMA Pediatrics*.

eTable 10. Incidence rates and hazard ratios with 95% confidence intervals for atrial fibrillation according to preterm birth and birth weight for gestational age, in births with information on maternal country of origin (N=6,382,972)

Exposure	Number of events	Rate, per 10,000 person-years	Crude HR (95% CI)	Adjusted HR (95% CI) [a]
**Preterm birth**				
**Overall follow-up**				
Term	10,074	0.70	1.00	1.00
Preterm	595	0.86	1.26 (1.16-1.37)	1.26 (1.14-1.39)
**First 18 years of follow-up (N=6,382,972)**				
Term	955	0.10	1.00	1.00
Preterm	110	0.24	2.37 (1.95-2.89)	2.45 (1.92-3.12)
**After 18 years of follow-up (N=4,031,030)**				
Term	9,119	1.76	1.00	1.00
Preterm	486	1.98	1.28 (1.14-1.43)	1.13 (1.02-1.26)
**Birth weight for gestational age**				
**Overall follow-up**				
SGA	1,205	0.74	0.96 (0.91-1.02)	0.93 (0.88-0.99)
AGA	8,088	0.67	1.00	1.00
LGA	1,377	0.94	1.53 (1.44-1.62)	1.54 (1.45-1.63)
**First 18 years of follow-up (N=6,382,972)**				
SGA	137	0.14	1.36 (1.13-1.63)	1.35 (1.13-1.63)
AGA	785	0.10	1.00	1.00
LGA	143	0.15	1.45 (1.21-1.73)	1.45 (1.21-1.74)
**After 18 years of follow-up (N=4,031,030)**				
SGA	1,068	1.66	0.93 (0.87-0.99)	0.90 (0.84-0.96)
AGA	7,303	1.70	1.00	1.00
LGA	1,234	2.52	1.54 (1.45-1.63)	1.55 (1.46-1.65)

Abbreviations: HR, hazard ratio; CI, confidence interval; SGA, small for gestational age; AGA, appropriate for gestational age; LGA, large for gestational age.

[a] We adjusted for country, calendar year of birth, maternal country of origin, parity, age, education level, marital status, hypertensive disorders and diabetes before childbirth. The model with preterm birth was further adjusted for the child's sex and birth weight.

© 2023 Yang F et al. *JAMA Pediatrics*.

eTable 11. Hazard ratios with 95% confidence intervals for atrial fibrillation according to preterm birth and birth weight for gestational age, after adjusting for the study participants' cardiometabolic diseases

Multivariate models	Adjusted HR (95% CI)		
	**Preterm vs. term birth**	**SGA vs. AGA birth**	**LGA vs. AGA birth**
**In the whole cohort (N=8,012,433)**			
Main model [a]	1.30 (1.18-1.42)	0.93 (0.88-0.99)	1.55 (1.46-1.63)
Further adjusted for hypertension [b]	1.29 (1.18-1.42)	0.92 (0.87-0.98)	1.55 (1.47-1.64)
Further adjusted for diabetes [c]	1.30 (1.18-1.43)	0.93 (0.88-0.99)	1.54 (1.46-1.63)
Further adjusted for IHD [d]	1.30 (1.18-1.43)	0.93 (0.87-0.98)	1.55 (1.46-1.63)
Further adjusted for stroke [e]	1.30 (1.18-1.43)	0.93 (0.88-0.99)	1.54 (1.46-1.63)
Further adjusted for heart failure [f]	1.30 (1.18-1.43)	0.92 (0.87-0.98)	1.55 (1.47-1.64)
**In individuals with information on BMI (N=1,112,290)**			
Main model [a]	1.25 (1.01-1.55)	0.84 (0.75-0.94)	1.39 (1.22-1.58)
Further adjusted for BMI [h]	1.25 (1.01-1.55)	0.84 (0.75-0.95)	1.38 (1.21-1.56)

Abbreviations: HR, hazard ratio; CI, confidence interval; SGA, small for gestational age; AGA, appropriate for gestational age; LGA, large for gestational age, IHD, ischemic heart disease; BMI, body-mass index.

[a] In the main model, we adjusted for country, calendar year of birth, maternal parity, age, education, marital status, hypertensive disorders and diabetes before childbirth. In case of analyses with SGA and LGA we also adjusted for sex and birth weight.
[b] We further adjusted for the study participants' hypertension in addition to the confounders in the main model. Of the 11,464 study participants who developed atrial fibrillation, 561 (4.9%) had a diagnosis of hypertension during follow-up.
[c] We further adjusted for the individual's diabetes in addition to the covariates in the main model. Of the 11,464 study participants who developed atrial fibrillation, 188 (1.6%) had diabetes during the follow-up.
[d] We further adjusted for the individual's IHD in addition to the covariates in the main model. Of the 11,464 study participants who developed atrial fibrillation, 179 (1.5%) had IHD during the follow-up.
[e] We further adjusted for the individual's stroke in addition to the covariates in the main model. Of the 11,464 study participants who developed atrial fibrillation, 150 (1.3%) had stroke during the follow-up.
[f] We further adjusted for the individual's heart failure in addition to the covariates in the main model. Of the 11,464 study participants who developed atrial fibrillation, 258 (2.3%) had heart failure during the follow-up.
[h] We further adjusted for the individual's body-mass index among individuals who have information on body-mass index in addition to the covariates in the main model.

© 2023 Yang F et al. *JAMA Pediatrics*.

eFigure 1. Flowchart of the study population

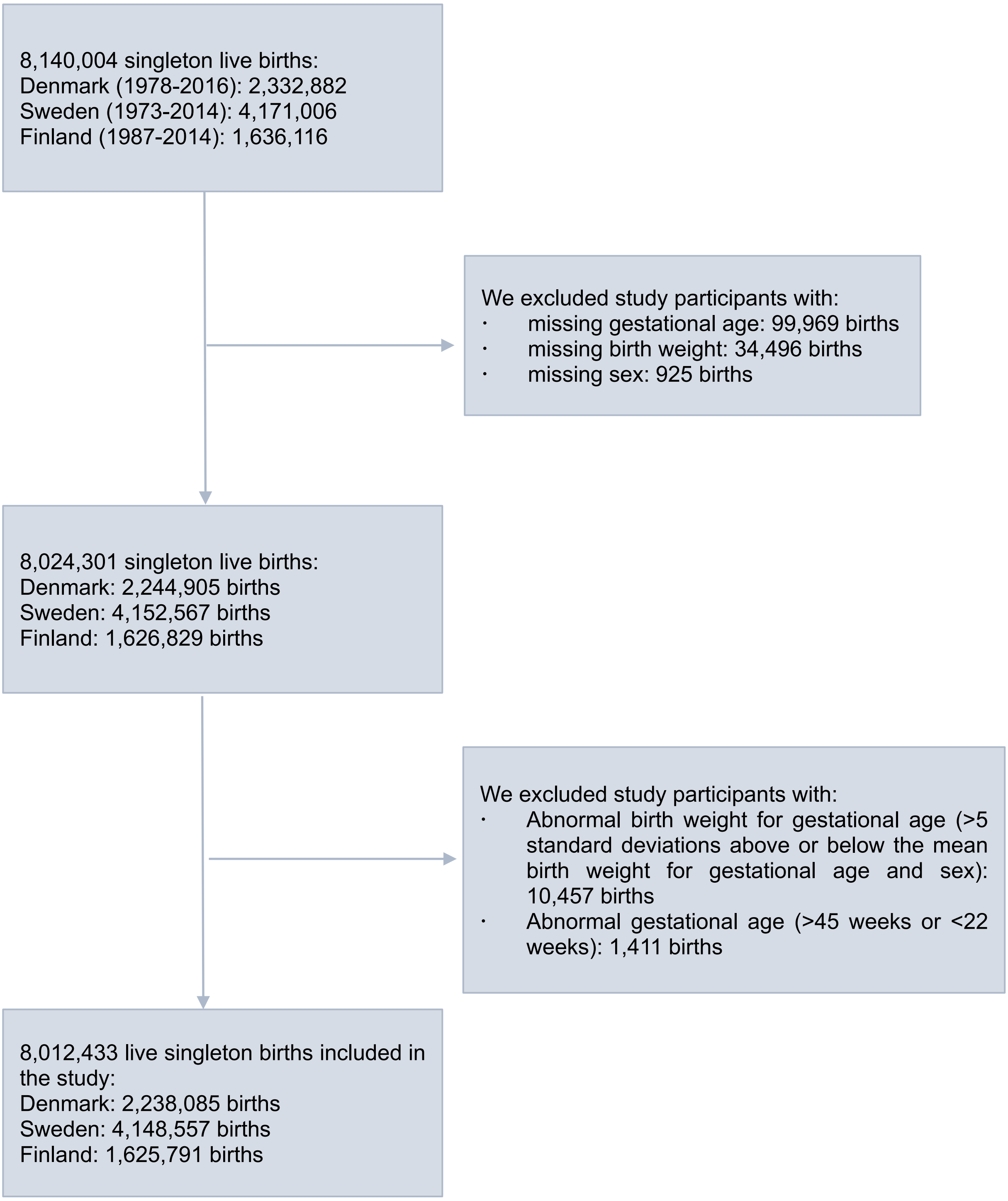

© 2023 Yang F et al. *JAMA Pediatrics*.

eFigure 2. Cumulative incidence of atrial fibrillation according to preterm birth and birth weight for gestational age

Preterm birth

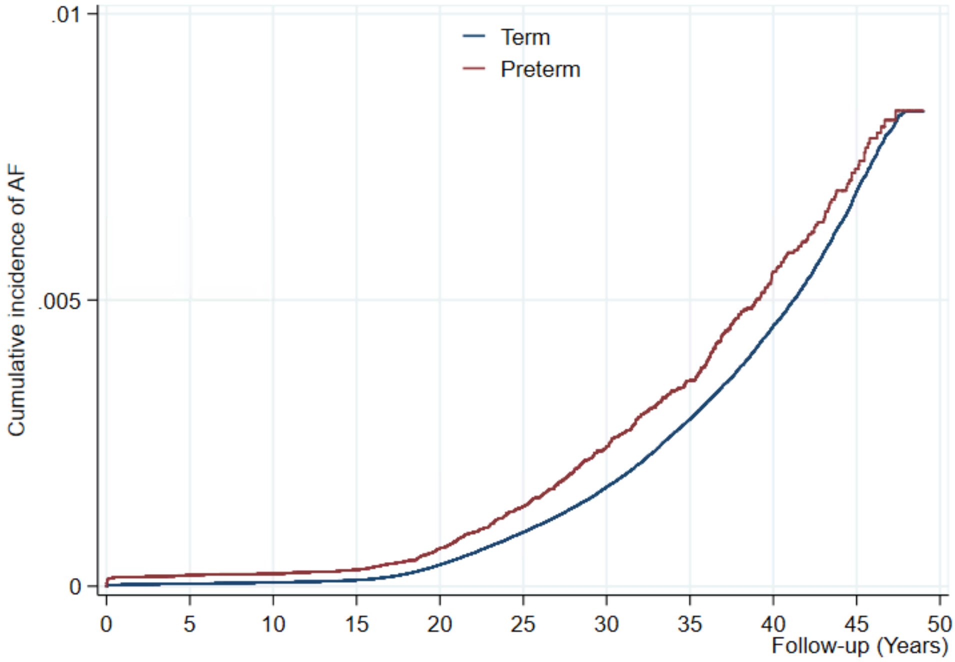

Number at risk

	0	5	10	15	20	25	30	35	40	45	50
Term	7633516	7001150	6097600	5044893	4025995	2977149	2013368	1269874	719380	334876	0
Preterm	378917	337044	294048	241621	191824	142353	96658	59706	31589	14320	0

Birth weight for gestational age

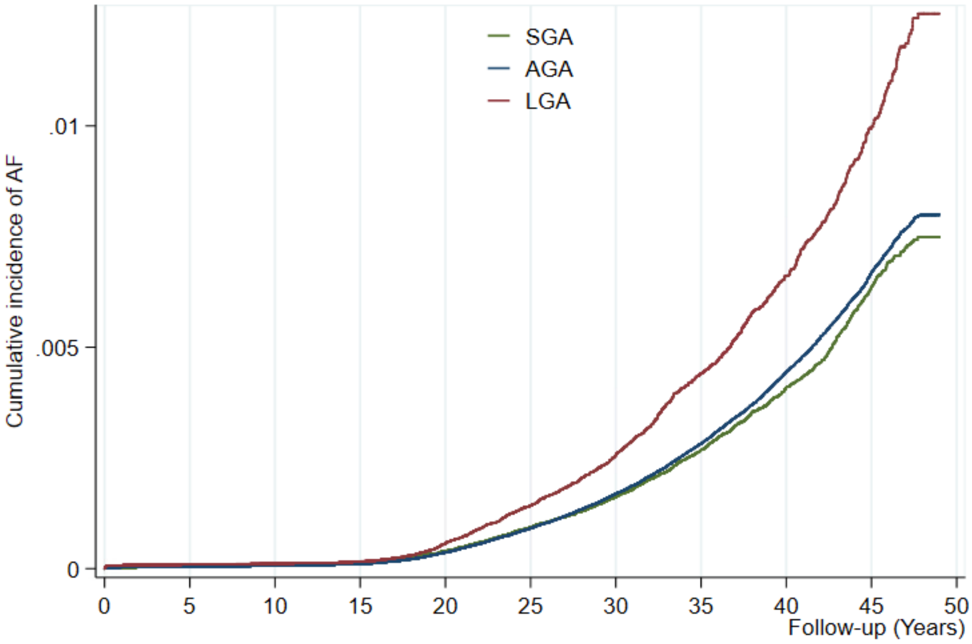

Number at risk

	0	5	10	15	20	25	30	35	40	45	50
SGA	800959	728241	640002	542198	447737	351386	256164	174440	101823	51689	0
AGA	6408715	5865263	5097967	4207403	3351416	2478303	1670135	1044263	589134	271114	0
LGA	802759	744690	653679	536913	418666	289813	183727	110877	60012	26393	0

Abbreviations: SGA, small for gestational age; AGA, appropriate for gestational age; LGA, large for gestational age.

© 2023 Yang F et al. *JAMA Pediatrics*.

eFigure 3. Adjusted hazard ratios and 95% confidence intervals for atrial fibrillation according to preterm birth and birth weight for gestational age, stratified by study country

Subgroup	Events, No.	Adjusted hazard ratio (95% CI)
**Preterm**		
**Overall follow-up years**		
Denmark	133	1.55 (1.25-2.94)
Sweden	463	1.20 (1.07-1.34)
Finland	53	1.90 (1.36-2.64)
**First 18 years of follow-up**		
Denmark	41	3.00 (2.01-4.48)
Sweden	69	2.18 (1.61-2.96)
Finland	22	2.02 (1.19-3.42)
**After 18 years of follow-up**		
Denmark	92	1.31 (1.01-1.70)
Sweden	394	1.11 (0.99-1.25)
Finland	31	1.82 (1.19-2.79)
**SGA**		
**Overall follow-up years**		
Denmark	280	1.03 (0.91-1.18)
Sweden	926	0.91 (0.85-0.98)
Finland	64	0.91 (0.71-1.18)
**First 18 years of follow-up**		
Denmark	56	1.56 (1.17-2.09)
Sweden	82	1.26 (0.99-1.60)
Finland	23	0.88 (0.57-1.36)
**After 18 years of follow-up**		
Denmark	224	0.95 (0.82-1.10)
Sweden	844	0.89 (0.82-0.95)
Finland	41	0.95 (0.69-1.31)
**LGA**		
**Overall follow-up years**		
Denmark	256	1.48 (1.29-1.69)
Sweden	1121	1.55 (1.45-1.65)
Finland	133	1.63 (1.35-1.97)
**First 18 years of follow-up**		
Denmark	53	1.50 (1.12-2.03)
Sweden	90	1.41 (1.12-1.77)
Finland	41	1.41 (1.01-1.98)
**After 18 years of follow-up**		
Denmark	203	1.47 (1.27-1.71)
Sweden	1031	1.56 (1.46-1.67)
Finland	92	1.75 (1.39-2.20)

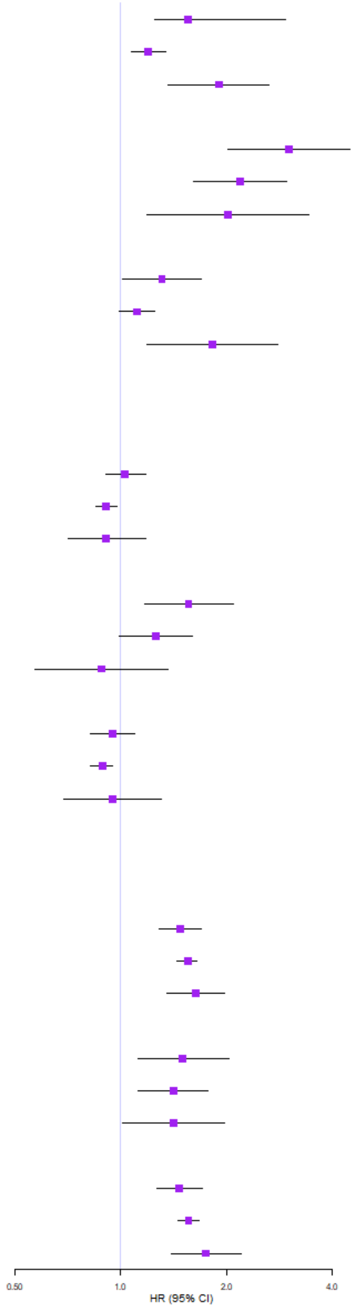

Abbreviations: HR, hazard ratio; CI, confidence interval; SGA, small for gestational age; AGA, appropriate for gestational age; LGA, large for gestational age.
We adjusted for country, calendar year of birth, maternal parity, age, education level, marital status, hypertensive disorders and diabetes before childbirth. The model with preterm birth was further adjusted for the child's sex and birth weight.

© 2023 Yang F et al. *JAMA Pediatrics.*

eAppendix 1. Description of the registers used in the study

1. The Danish registers

- The Danish Civil Registration System

  The Danish Civil Registration System was established in 1968, and includes all individuals living in Denmark.[1] We used this register to extract data on gender, birth date, place of birth, vital status, identity of parents and siblings, marital status, emigration and immigration.
- The Danish Integrated Database for Labour Market Research

  The Danish Integrated Database for Labour Market Research started in 1980, and provided us information on gender, age and education.[2]
- The Danish Medical Birth Register

  The Danish Medical Birth Register was established in 1973 and includes data on virtually all births in Denmark.[3] From this register, we extracted information on maternal characteristics such as height and weight prior to conception (available since 2003), smoking in early pregnancy (available since 1991), age at delivery and parity, characteristics of the newborn such as singleton/multiple birth, birth date, sex, gestational age, birth weight and length at birth.
- The Danish National Patient Register

  The Danish National Patient Register holds information on all somatic hospitalizations in Denmark since 1977, while specialized outpatient care and emergency department contacts were included since 1995.[4] Diagnoses were coded using the Danish version of International Classification of Diseases, version 8 (ICD-8) up to 1995 and ICD-10 afterwards. This register also holds information on surgical procedures, which have been coded using the Danish Classification of Surgical Procedures during 1977-1996 and using the Danish version of the Nordic Medico-Statistical Committee Classification of Surgical Procedures in later years. We retrieved from this register information on the diseases of interest and on cardiac surgery.
- The Register of Causes of Death

  The Danish Register of Cause of Death was established in 1875; individual death records have been computerized since 1970.[5] We extracted information on date of death and cause of death for index persons and their relatives from the Register of Cause of Death. The ICD-8 was used to classify the causes of death during 1970-1993, whereas the ICD-10 was used from 1994 onward.

2. The Swedish registers

- The Swedish Total Population Register

  The Swedish Total Population Register contains data on Swedish residents since 1968.[6] We collected information on the date of birth, place of birth, sex, civil status, marital status, dates of immigration and emigration.
- The Swedish Multi-Generation Register

  The Swedish Multi-generation register contains information on child-parent relationships for index persons born since 1932.[7] This register enabled us to identify children, siblings, parents, grandparents, etc. for cohort members and their mothers.
- The Swedish Register of Education

  The Swedish Register of Education was established in 1985 and contains yearly updated information on Swedish residents aged 16-74 years. We retried from this register information on the mother's highest completed educational attainment.[8]
- The Swedish Medical Birth Register

  The Swedish Medical Birth Register was established in 1973. We obtained information on maternal age at delivery, parity, complications during pregnancy, child's sex, birth date, singleton/multiple birth, birth weight, and gestational age. We also included data on maternal smoking, height, pre-pregnancy weight and weight gain during pregnancy (available since 1982).[9] Complications during pregnancy were coded according to the Swedish version of the ICD; ICD-8 was in use until 1986, ICD-9 from 1987 to 1996, and ICD-10 since 1997.
- The Swedish Patient Register

  The Swedish Patient Register contains information on inpatient care since 1964 and its coverage became nationwide in 1987; outpatient hospital care was included since 2001.[10] From this register we included the date and time of inpatient admission or outpatient treatment, primary and secondary diagnoses for each hospital contact.
- The Swedish Cause of Death Register

  The Swedish Cause of Death Register is a high quality virtually complete register of all deaths in Sweden since 1952.[11] We obtained data on date and cause of death from this register.

© 2023 Yang F et al. *JAMA Pediatrics*.

3. The Finnish registers

- The Finnish Central Population Register

  The Finnish Central Population Register was created in 1968 and covers basic information on all citizens and permanent residents in Finland.[12] From this register we retrieved individual information on gender, date of birth, death, marital status, and immigration and emigration.
- The Education Register at Statistics Finland

  The Statistics Finland started in 1865, and is the only public authority created specifically for statistical services in Finland.[13] We used data from the Education Register, kept by Statistics Finland, to ascertain the data on maternal highest education before delivery.
- The Finnish Medical Birth Register

  The Finnish Medical Birth Register was established in 1987.[14] This register provided data on maternal smoking in early pregnancy, age at delivery and parity, singleton/multiple birth, birth date, sex, gestational age, and birth weight.
- The Finnish Hospital Discharge Register

  The Finnish Hospital Discharge Register contains information from all inpatient wards since 1967 and all outpatient visits to public hospitals since 1998.[15] We used this register to obtain dates of inpatient admission and outpatient treatment, and primary and secondary diagnoses at discharge for each individual included in our study. The diagnoses were coded according to the Finnish version of ICD-9 until 1995 and ICD-10 from 1996 onwards.

© 2023 Yang F et al. *JAMA Pediatrics*.

eAppendix 2. Description of the source, the measurement and the categorization of covariates

Information on maternal characteristics including age at delivery (≤19, 20-24, 25-29, 30-34, and ≥35 years), parity (1, 2, ≥3), and body-mass index (BMI, available only in Denmark and Sweden) and smoking in early pregnancy (yes vs. no), and the child's characteristics including calendar year of birth (1973-1978, 1979-1984, 1985-1990, 1991-1996, 1997-2002, 2003-2008, 2009-2016), singleton/multiple birth, and sex (boy or girl) was obtained from the Medical Birth Registers in Denmark, Sweden and Finland. Maternal BMI was further classified as underweight (BMI <18.5 kg/m^2), normal weight (18.5–24.9 kg/m^2), overweight (25.0–29.9 kg/m^2), and obesity (≥30 kg/m^2).

Information on maternal country of origin (same as the country where the birth is registered versus not) and marital status before delivery (married/registered partnership versus not) was obtained from the Danish Civil Registration System, the Swedish Total Population Register, and the Finnish Central Population Register. Data on maternal education before delivery was extracted from the Danish Integrated Database for Labor Market Research, the Swedish Register of Education and Statistics Finland.

To retrieve information on maternal family history of cardiovascular diseases before delivery, we used the Danish Civil Registration System and the Swedish Multi-generation register to identify the mother's family members. We extracted information on these relatives' cardiovascular diseases diagnoses (ICD-8/9: 390–459, ICD-10: I00-I99) from the Danish National Patient Register (DNPR), the Danish Register of Cause of Death, the Swedish Patient Register (SPR) and the Swedish Cause of Death Register (SCDR).

We retrieved data on maternal diabetes before delivery, i.e., pregestational diabetes and gestational diabetes mellitus, from the DNPR, the Swedish Medical Birth Register (SMBR) and from the Finnish Hospital Discharge Register (FHDR). Maternal diabetic disorders were identified using the following ICD codes: 1) in Denmark: ICD-8: 249, 250; ICD-10: E10-E14, O24; 2) in Sweden: ICD-8: 250; ICD-9: 250, 648A, 648W; ICD-10: E10-E14, O24; 3) in Finland: ICD-9: 250, 6480A, 6488A; ICD-10: E10-E14, O24.

Maternal hypertensive disorders during pregnancy including pre-existing chronic hypertension, gestational hypertension, preeclampsia, eclampsia, and HELLP syndrome (hemolysis, elevated liver enzymes, low platelet count) were also extracted from the DNPR, SMBR and FHDR. The used ICD codes are 1) in Denmark: ICD-8: 40009, 40019, 40029, 40039, 40099, 40199, 63700, 63703, 63704, 63709, 63719, 76029; ICD-10: I10, I11, I12, I13, I15, O10, O11, O13, O14, O15, O16; 2) in Sweden: ICD-8: 400, 401, 402, 403, 404, 63701, 63703, 63704, 63709, 63799, 63710; ICD-9: 401, 402, 403, 404, 405, 642A, 642B, 642C, 642D, 642E, 642F, 642G, 642H, 642X; ICD-10: I10, I11, I12, I13, I15, O10, O11, O13, O14, O15, O16; 3) in Finland: ICD-9: 4019, 4029, 4039, 4040, 4059, 6420, 6421, 6422, 6423, 6424, 6425, 6426, 6427, 6429; ICD-10: I10, I11, I12, I13, I15, O10, O11, O13, O14, O15, O16.

Data on the study participant's BMI was available from the Danish and Swedish MBR, only for women who later became pregnant. Data on the study participant's cardiac surgery was identified from the DNPR using the Danish version of the surgical procedures (30,31, KFA-KFX).

Data on the study participants' congenital anomalies was obtained from the DNPR, SPR and FHDR using the ICD codes: 1) in Denmark: ICD-8: 740-759 and ICD-10: Q00-Q99; 2) in Sweden: ICD-8/9: 740-759 and ICD-10: Q00-Q99; 3) in Finland: ICD-9: 740-759 and ICD-10: Q00-Q99.

Data on the study participants' hypertension was obtained from the DNPR, SPR and FHDR using the ICD codes: 1) in Denmark: ICD-8: 400-404 and ICD-10: I10-I15, O10, O11; 2) in Sweden: ICD-8: 400-404; ICD-9: 401-405, 642A, 642B, 642C, 642H; and ICD-10: I10-I15, O10, O11; 3) in Finland: ICD-9: 4019, 4029, 4039, 4040, 4059, 6420, 6421, 6422, 6427; and ICD-10: I10-I15, O10, O11.

Data on the study participants' diabetes was obtained from the DNPR, SPR and FHDR using the ICD codes: 1) in Denmark: ICD-8: 250 and ICD-10: E10-E14; 2) in Sweden: ICD-8: ICD-8/9: 250 and ICD-10: E10-E14; 3) in Finland: ICD-9: 250 and ICD-10: E10-E14.

Data on the study participants' ischemic heart disease was obtained from the DNPR, SPR and FHDR using the ICD codes: 1) in Denmark: ICD-8: 410-414 and ICD-10: I20-I25; 2) in Sweden: ICD-8/9: 410-414 and ICD-10: I20-I25; 3) in Finland: ICD-9: 4100, 4109, 4110, 4120, 4121, 4131, 4140, 4148, 4149; and ICD-10: I20-I25.

Data on the study participants' stroke was obtained from the DNPR, SPR and FHDR using the ICD codes: 1) in Denmark: ICD-8: 430, 431, 433, 434, 436 and ICD-10: I60, I61, I63, I64; 2) in Sweden: ICD-8/9: 430, 431, 433, 434, 436 and ICD-10: I60, I61, I63, I64; 3) in Finland ICD-9: 430, 431, 433, 434, 436 and ICD-10: I60, I61, I63, I64.

Data on the study participants' heart failure was obtained from the DNPR, SPR and FHDR using the ICD codes: 1) in Denmark: ICD-8: 42709, 42710, 42711, 42719 and ICD-10: I110, I130, I132, I50;

© 2023 Yang F et al. *JAMA Pediatrics*.

2) in Sweden: ICD-8: 427.00, 427.10; ICD-9: 428 and ICD-10: I110, I130, I132, I50; 3) in Finland: ICD-9: 428 and ICD-10: I110, I130, I132, I50.

© 2023 Yang F et al. *JAMA Pediatrics*.

eReferences

1. Pedersen CB. The Danish civil registration system. *Scandinavian journal of public health*. 2011;39(7_suppl):22-25.
2. Petersson F, Baadsgaard M, Thygesen LC. Danish registers on personal labour market affiliation. *Scandinavian journal of public health*. 2011;39(7_suppl):95-98.
3. Knudsen LB, Olsen J. The Danish Medical Birth Registry. *Danish medical bulletin*. 1998;45(3):320-323.
4. Lynge E, Sandegaard JL, Rebolj M. The Danish national patient register. *Scandinavian journal of public health*. 2011;39(7_suppl):30-33.
5. Helweg-Larsen K. The Danish register of causes of death. *Scandinavian journal of public health*. 2011;39(7_suppl):26-29.
6. Ludvigsson JF, Almqvist C, Bonamy A-KE, et al. Registers of the Swedish total population and their use in medical research. *European journal of epidemiology*. 2016;31(2):125-136.
7. Ekbom A. The Swedish multi-generation register. *Methods in biobanking*. Springer; 2011:215-220.
8. Ludvigsson JF, Svedberg P, Olén O, Bruze G, Neovius M. The longitudinal integrated database for health insurance and labour market studies (LISA) and its use in medical research. *European journal of epidemiology*. 2019;34(4):423-437.
9. Health NBo, Welfare. The Swedish Medical Birth Register–A summary of content and quality. National Board of Health and Welfare Stockholm, Sweden; 2003.
10. Ludvigsson JF, Andersson E, Ekbom A, et al. External review and validation of the Swedish national inpatient register. *BMC public health*. 2011;11(1):1-16.
11. Brooke HL, Talbäck M, Hörnblad J, et al. The Swedish cause of death register. *European journal of epidemiology*. 2017;32(9):765-773.
12. Haukka J. Finnish health and social welfare registers in epidemiological research. *Norsk epidemiologi*. 2004;14(1)
13. Chudal R, Sucksdorff D, Suominen A, et al. Finnish Prenatal Study of Bipolar Disorders (FIPS-B): overview, design and description of the sample. *Nordic journal of psychiatry*. 2014;68(3):169-179.
14. Teperi J. Multi method approach to the assessment of data quality in the Finnish Medical Birth Registry. *Journal of Epidemiology & Community Health*. 1993;47(3):242-247.
15. Sund R. Quality of the Finnish Hospital Discharge Register: a systematic review. *Scandinavian journal of public health*. 2012;40(6):505-515.

© 2023 Yang F et al. *JAMA Pediatrics*.
