# Supplementary material for: Preterm Birth, Small for Gestational Age, and Large for Gestational Age and the Risk of Atrial Fibrillation Up to Middle Age
Source: JAMA Pediatr. 2023 Apr 24;177(6):599–607. doi: 10.1001/jamapediatrics.2023.0083 (PMC10126943; doi:10.1001/jamapediatrics.2023.0083)
Supplement: Supplement 2. — Data sharing statement [file jamapediatr-e230083-s002.pdf]

Data Sharing Statement

Yang. Preterm Birth, Small for Gestational Age, and Large for Gestational Age and the Risk of Atrial Fibrillation Up to Middle Age. *JAMA Pediatr*. Published April 24, 2023. doi:10.1001/jamapediatrics.2023.0083

Data
Data available: No

Additional Information
Explanation for why data not available: No data from national registers is allowed to be shared with the third party.
